# Virulence factors of *Streptococcus pyogenes* strains from women in peri-labor with invasive infections

**DOI:** 10.1007/s10096-016-2593-0

**Published:** 2016-02-12

**Authors:** E. Golińska, M. van der Linden, G. Więcek, D. Mikołajczyk, A. Machul, A. Samet, A. Piórkowska, M. Dorycka, P. B. Heczko, M. Strus

**Affiliations:** Jagiellonian University Medical College, 18 Czysta Str., 31-121 Kracow, Poland; Institute of Microbiology, RWTH Aachen University, Faculty of Medicine and University Hospital, Aachen, Germany; Department of Clinical Microbiology, University Clinical Center in Gdansk, 7 Debinki Str., 80-952 Gdansk, Poland; Microbiological Laboratory, Diagnostics Inc., Kracow Branch, Na Skarpie 66 axis, 31-913 Kracow, Poland

## Abstract

Invasive group A streptococcal (GAS) infections constitute an important epidemiological problem. Many cases occur in women during the postnatal period. The objective of this study was to evaluate the presence of the genes responsible for production of iron-chelating protein (perR) and superantigens (speA, speB, speC, speF, speG, speH, speI, speJ, speK, speL, speM, smeZ, and ssa) in S. pyogenes strains isolated from invasive infections in women after delivery. Furthermore, this study sought to verify whether S. pyogenes strains show special phenotypic and genotypic (sla, spy1325) characteristics that may play a decisive role in adherence to the genital tract epithelium. Moreover, the emm-types and antibiotic susceptibility were determined. We tested 30 invasive S. pyogenes strains isolated from postpartum invasive infection and 37 GAS control strains isolated from the genital tracts of asymptomatic multiparous women. The majority of the tested strains were shown to express two types of emm genes (1 and 28), though emm −12, −28, −75 and −89 were uniquely expressed in the group of strains isolated from invasive infections. A significantly higher prevalence of perR in the strains from puerperal fever was shown. Significant differences were also found between the two groups with respect to the incidence of the genes related to adherence; GAS strains originating from women with sepsis/puerperal fever showed presence of these genes less frequently than those of the control group. Although differences in frequencies of the gene coding for various superantigens were noted between the compared groups of GAS strains, they were not significant.

## Introduction

Group A streptococci (GAS) have been recognized as one of the leading infectious agents in human puerperal fever. GAS have also been implicated in several more localized infections affecting the throat, skin, ear, sinuses, or vagina. Invasive infections such as streptococcal toxic shock syndrome and sepsis began to receive attention from epidemiologists only quite recently, when the United States, Norway, Sweden, and Denmark reported higher incidence rates, outbreaks, and even epidemics [1]. More importantly, systemic *S. pyogenes* infections commonly result in high mortality. The number of such infections is on a constant rise, especially in developed countries, and may pose a serious diagnostic and/or therapeutic problem [2, 3].

GAS strains express many virulence factors including surface protein M, streptolysins, streptokinase, hyaluronidase, peptidoglycan, and teichoic acid. Protein M is considered as the main virulence factor, limiting phagocytosis, disturbing the function of complement, and being responsible for adhesion [4]. Moreover, invasive *S. pyogenes* strains, which cause deep soft-tissue infections and fasciitis as well as streptococcal toxic shock syndrome (STSS) and sepsis, also produce highly specific toxins with special pro-inflammatory properties. Streptococcal pyrogenic toxins (SPE) possess superantigen properties and bridge antigen-presenting cells with immune system effector cells, leading to their polyclonal activation. This activation leads to accelerated T lymphocyte proliferation and liberation of significant quantities of pro-inflammatory cytokines, leading to toxic shock [5]. Furthermore, the *emm* gene forms the basis for epidemiological typing of GAS at the same time correlating serotyping with pathogenicity [6].

An intriguing phenomenon observed by epidemiologists worldwide is the rise in the incidence of invasive *S. pyogenes* infections in women in labor and childbirth, especially after caesarean delivery [7, 8]. Studies have shown that GAS infections in women in labor are most commonly caused by strains belonging to the emm28 serotype, or less commonly, emm1 [9]. Based on other publications, the hyper-invasiveness of *S. pyogenes* was shown to be the result of the presence of surface protein *spy1325*, which allows GAS strains to strongly adhere to the host cell surface. The recently discovered *SpyCEP* protease, which is able to digest IL-8, may result in a significant decrease in host phagocytic cell activity [4, 10].

All of these mechanisms, collectively defining the invasiveness of GAS, do not fully explain the strong affinity of *S. pyogenes* strains for the female genital tract, especially during labour [3]. It is probable that labor and delivery, especially by a caesarean section (CC), increases the amount of freely available blood in the uterus and other compartments and may lead to an increase in the size of the populations of *S. pyogenes*, possibly producing both the superantigens as well as other virulence proteins.

The main objective of this study was to evaluate the relationship between the gene encoding production of peroxide stress response and metal-binding regulator protein (*perR*), and the genes encoding superantigens (*speA, speB, speC, speF, speG, speH, speI, speJ, speK, speL, speM, smeZ*, and *ssa*) in the development of invasive GAS disease. Furthermore, this study sought to verify whether S. pyogenes strains isolated from women with puerperal fever and/or postpartum sepsis show special phenotypic and genotypic (presence of *sla* and *spy1325* genes) characteristics relating to enhanced adherence to the genital tract epithelium. Moreover, the emm-type and antibiotic susceptibility to penicillin G, erythromycin, and clindamycin were determined.

## Materials and methods

The study material consisted of 30 GAS strains isolated from women in labor presenting with clinical symptoms of puerperal fever and postpartum sepsis, as defined by CDC [11]. In this group, 12 *S. pyogenes* strains originated from tissues of women with puerperal fever (including two patients with necrotizing fasciitis) and 18 strains were isolated from blood of patients with sepsis (including four cases with streptococcal toxic shock syndrome). The control group comprised 37 GAS strains isolated from the genital tract of non-symptomatic, non-pregnant women aged 18–50 years who were diagnosed with abnormal vaginal microbiota. All strains were isolated in medical facilities across Northern and South-Eastern Poland and shipped to Jagiellonian University Medical College in Krakow, or across Germany and shipped to the University of Aachen in Germany.

Speciation of the isolates was performed using phenotypic methods (API, bioMérieux, France) and the latex agglutination test for serological grouping of β-haemolysing streptococci (Oxoid, UK). In the case of inconclusive results, PCR was performed with species-specific primers *spy1258F* and *spy1258R* aimed at the transcriptional regulator gene *spy1258*. Primers used in the reactions were those described elsewhere [3, 12–18]. Amplification was performed according to the methodology described by LIu et al. [12].

### Antibiotic susceptibility testing

Antibiotic susceptibility to penicillin G, erythromycin, and clindamycin was determined by the disk diffusion method on Müller–Hinton agar with 5 % sheep blood according to European Committee on Antimicrobial Susceptibility Testing (EUCAST) guidelines (http://www.eucast.org/antimicrobial_susceptibility_testing/calibration_and_validation/).

### Detection of *emm* gene serotype by PCR

The presence of the *emm* gene was verified by PCR as described by Podbielski et al. [8, 13]. The PCR products were sequenced using an ABI Prism® 310 Genetic Analyzer (Applied Biosystems, Weiterstadt, Germany). The sequences obtained were compared with all available reference sequences on the United States (US) Center for Disease Control (CDC) website (http://www2a.cdc.gov/ncidod/biotech/strepblast.asp). The *emm* gene was considered present if the degree of identity between the sequences reached 95 %.

### PCR-based gene detection

Presence or absence of the gene encoding *PerR*, genes coding for different serotypes of SPE and the genes responsible for adherence (*sla, spy1325*) was evaluated using PCR (for *speI, speJ, speK, speL, speM, smeZ, sla, spy1325*,and *perR*) and multiplex PCR (for *speA, speB, speC, speF, speG, speH*, and *ssa)*. Primers used in the reactions were those described elsewhere [3, 12–18]. Amplification was performed according to methodologies previously described [3, 14–18].

### Adherence of *S. pyogenes* strains to human endometrial cell line HEC 1B

Human endometrial cell line HEC 1B was used for *S. pyogenes* adherence studies. The cells were cultured in 24-well plastic cell culture plates (TPP, Switzerland) with 3.5 ml of Dulbecco's modified Eagle's medium (DMEM) supplemented with 10 % fetal bovine serum (FBS) and 1 % penicillin. The cultures were maintained at 37 °C and 5 % CO2, and the culture medium was refreshed every 24 h. The cultures were continued until a monolayer of approximately 1 × 106 cells/ml was obtained. Then the cells were harvested by trypsin detachment and transferred into wells of the next plates with round coverslips on the bottom. The cells were grown in the same medium without antibiotic for 72 hours to obtain a monolayer culture. The *S. pyogenes* adherence test was performed by adding 100 μl of bacterial culture containing 1 × 108 colony-forming units (CFU)/ml to HEC 1B cells. The initial, bacteria to cells, ratio was 100:1. The plates containing cells and bacteria were incubated under aerobic conditions at 37 °C and 5 % CO2 for 2 h to allow bacteria to adhere. After this time, the liquid medium was removed and the plates washed twice with 37 °C PBS. The bacteria adhered to the cells were stained by Gram’s method. Each specimen was analyzed using a light microscope, under 1,000 × magnification. The number of adherent microbial cells to tissue cultures was the arithmetical average of counts from five microscopic fields. The adherence ability of studied bacteria was evaluated using a semi-quantitative scale from 0–3, as used by us before [19] and based on the following legend:

- strong adherence (3): > 150 bacterial cells per field
- moderate adherence (2): 100–150 bacterial cells per field
- weak adherence (1): 40–100 bacterial cells per field
- no adherence (0): < 40 bacterial cells per field

All experiments were run in duplicate.

### Statistics

Statistical analyses were performed to demonstrate significant differences in the presence of selected genes and in the levels of adherence abilities among tested GAS strains. The Fisher exact test was used for the analysis of gene occurrence, while the Mann–Whitney–Wilcoxon test was used to compare adherence data.

## Results

Assays for susceptibility to penicillin G, erythromycin, and clindamycin were performed on 67 GAS strains. All strains showed phenotypic susceptibility to penicillin. Five strains from the control group showed resistance phenotypes to erythromycin and clindamycin. Resistance profiles and detailed characteristics of *S. pyogenes* strains are presented in Table 1.

**Table 1:** Characteristics of the GAS strains studied

Characteristics	No of strains isolated from postpartum invasive infection	No of strains isolated from vaginal carriage
Serogrouping: group A	30	37
Phenotyping (API Strep): S.pyogenes	30	37
Sensitive to penicillin	30	37
MLSB resistance profile (erytromycin 15 ug/ + clindamycin 2 ug)	0	5

Typing of the M protein was performed on all strains. The results (Table 2) show that, among strains isolated from invasive infections, *emm* type 28 predominated *emm* type 1 and other types. Eight different isotypes were detected among control strains, with isotype 28 being dominant and followed with types 1 and 2. There were no significant differences between these two groups of the strains in frequency of *emm* types distribution.

**Table 2:** Determinations of adherence level and distribution of different emm serotypes in the genome of *Streptococcus pyogenes* strains from

a) Invasive groups				
Number of isolates	Test group	emm type	% of emm types from test groups	Adherence level to HEC 1B tissue
1	puerperal fever	28	36,6 %	2
2	puerperal fever	28		3
3	puerperal fever	28		1
4	puerperal fever	28		2
5	puerperal sepsis	28		1
6	puerperal sepsis	28		2
7	puerperal sepsis	28		3
8	puerperal sepsis	28		1
9	puerperal sepsis	28		3
10	puerperal sepsis	28		1
11	puerperal fever/necrotizing fasciitis	28		2
12	puerperal fever	1	23,3 %	3
13	puerperal sepsis	1		3
14	puerperal sepsis	1		1
15	puerperal sepsis/STSS	1		2
16	puerperal sepsis/STSS	1		2
17	puerperal sepsis/STSS	1		1
18	puerperal sepsis/STSS	1		2
19	puerperal fever	12	10,0 %	3
20	puerperal fever	12		0
21	puerperal sepsis	12		2
22	puerperal sepsis	77	10,0 %	0
23	puerperal sepsis	77		1
24	puerperal sepsis	77		1
25	puerperal fever	89	10,0 %	3
26	puerperal sepsis	89		0
27	puerperal fever/necrotizing fasciitis	89		0
28	puerperal fever	2	6,6 %	1
29	puerperal fever	2		3
30	puerperal sepsis	75	3,3 %	2
b) Control group				
Number of isolates	Test group	emm type	% of emm types from control groups	Adherence level to HEC 1B tissue
1	control	28	18,9 %	1
2	control	28		0
3	control	28		1
4	control	28		3
5	control	28		1
6	control	28		0
7	control	28		3
8	control	1	16,2 %	1
9	control	1		2
10	control	1		2
11	control	1		0
12	control	1		1
13	control	1		1
14	control	2	13,5 %	1
15	control	2		2
16	control	2		2
17	control	2		2
18	control	2		2
19	control	12	8,1 %	3
20	control	12		2
21	control	12		3
22	control	89	8,1 %	3
23	control	89		2
24	control	89		3
25	control	3	5,4 %	3
26	control	3		1
27	control	77	5,4 %	3
28	control	77		3
29	control	4	2,7 %	2
30	control	not analysable	21,6 %	2
31	control	not analysable		3
32	control	not analysable		2
33	control	not analysable		2
34	control	not analysable		3
35	control	not analysable		2
36	control	not analysable		2
37	control	not analysable		3

Legend:

Adherence level — a) strong adherence (3): >150 bacterial cells per field

b) moderate adherence (2): 100–150 bacterial cells per field

c) weak adherence (1): 40–100 bacterial cells per field

d) no adherence (0): < 40 bacterial cells per field

**Fig. 1:**
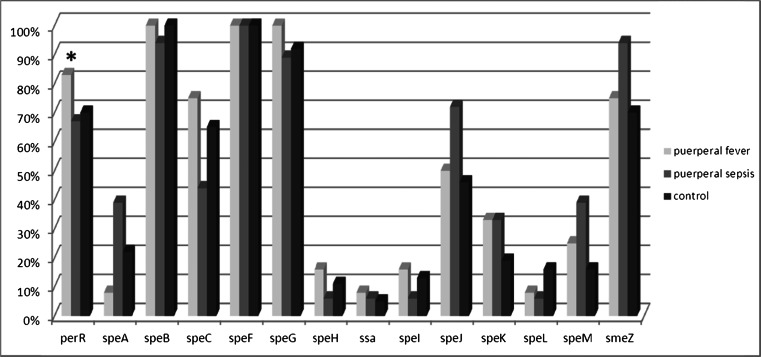
Proportional occurrence of virulence genes in *Streptococcus pyogenes* strains (* =  *p* < 0.05)

Examination for the presence of the virulence genes *perR, speA, speB, speC, speF, speG, speH, speI, speJ, speK, speL, speM, smeZ, ssa, sla*, and *spy1325* was performed for all 67 strains (Fig. 1). This covered all genes responsible for the synthesis of pyrogenic exotoxins [14, 15, 18], and *sla* and *spy1325* genes, which control adherence [3, 16].

The *perR* gene was detected in the genome of ten of 12 GAS isolates from women with puerperal fever (83 %), and this value showed a statistically significant difference (*p* < 0.05) in comparison to the control group (26 of 37 strains; 70 %) (Fig. 1). Such a statistically significant difference was not confirmed between the group of the streptococci isolated from women with sepsis and those of the control group. Moreover, no recurring relationship was found between the presence of a given type of protein M and the absence of the *perR* gene. The absence of the *perR* gene was shown in the GAS genome of strains with *emm* gene serotypes 28 and 89 in the group of women with puerperal fever and with *emm* gene types 1, 12, 28, and 77 in patients with sepsis.

By analyzing the presence of the genes encoding the different exotoxin types in bacterial DNA, *speF, speG*, and *speB* were found to be present in nearly all GAS strains independent of the group studied; however, *speH, ssa, speI,* or *speL* were rare and present only in single cases, either in GAS from patients with invasive infections or in the control group. *SpeA, speJ, speK, speM*, and *speJ* were detected in the specific groups with varied frequency. These differences were not statistically significant (*p *> 0.05). However, *speM* and *smeZ* were present more commonly in the isolates from the women with sepsis (39 % and 94 % respectively) in comparison to strains from patients with puerperal fever (25 % and 75 % respectively) or the control group (16 % and 70 % respectively). Also, *speK* was nearly twice as common in the isolates from septic women (33 %) and those with puerperal fever (33 %) as in the control group strains (19 %). Similarly increased presence of *speJ* and *speA* was shown in GAS strains isolated from septic women compared to puerperal fever and control groups. Furthermore, in genomic DNA of S. pyogenes strains isolated from women with streptococcal sepsis, the most common genes responsible for synthesis of pyrogenic exotoxins were *speZ, speJ, speA*, and *speM*.

**Fig. 2:**
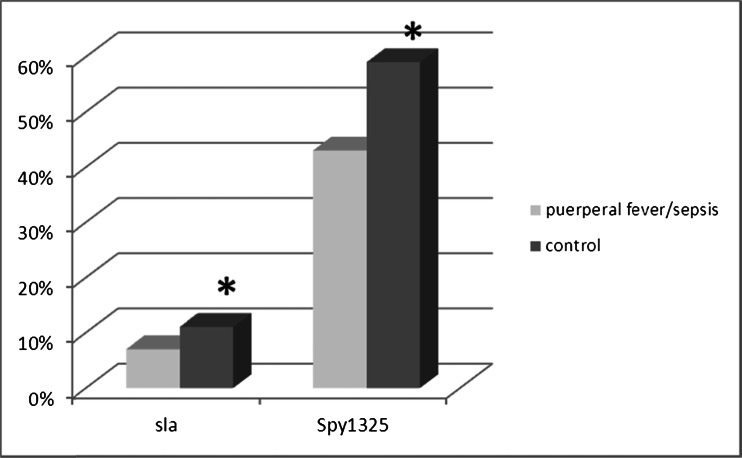
Proportional occurrence of genes related to adherence (*sla* and *spy1325*) in the genome of studied Streptococcus pyogenes strains (* =  *p* < 0.05)

On the other hand, it appeared that the genes *sla* and *spy1325* were significantly more frequent in the GAS from women in the control group rather than from patients with any invasive form of GAS infection (Fig. 2). The same statistically significant difference (*p* < 0.05) was found when studying the phenotypic characteristics of the adherence of 67 GAS to HEC 1B cells strains. Again, a much stronger adherence to host cells was observed in GAS isolated from women in the control group than from the other two groups (Table 1).

## Discussion

Group A streptococci have the capacity to breach epithelial barriers and cause a variety of invasive diseases. While it is conceivable that tissue site preferences for infection in the throat or skin might be explained by the expression and/or regulation of tissue-specific colonization factors such as adhesins, the exact mechanisms responsible for tissue tropism in the genital tract are yet to be elucidated [4].

Detailed epidemiological data from European countries demonstrate an increasing incidence of severe invasive GAS infections all over the continent, also related to labor. [20].

In the current study, we observed various *emm* gene serotypes in GAS strains isolated from the control group and from patients with invasive infections. The domination of *emm1* and *emm28* serotypes in GAS strains isolated from recent outbreaks has been reported by Plainvert et al. [21], Lamagni et al. [1], and Strus et al. [8].

Changes in the epidemiology of *S. pyogenes* have drawn the attention of researchers towards various virulence factors of these bacteria, mainly the Fe2+ binding proteins, pyrogenic exotoxins (*SpeA-M, SmeZ*), streptococcal superantigens (*Ssa*), and protein M. These virulence factors play a major role in the development and pathogenesis of the invasive infections [22–24].

Iron, an essential nutrient for most bacterial pathogens, functions as a catalyst in electron transport systems and is an important cofactor in many essential enzymes. There are many reports in the literature about enhanced invasiveness and faster growth of different bacteria, both Gram-positive as well as Gram-negative, which possess iron-binding mechanisms [25–27]. Among different GAS proteins involved in iron ion acquisition, the gene *perR* encoding the peroxide resistance regulator has been shown to be important for GAS fitness within the host and necessary for resistance to phagocytic killing in human blood. *PerR* gene has also recently been reported to enhance GAS oxidative stress resistance and virulence in the host [28, 29]. Thus, PerR and other related proteins are considered to be among the major GAS virulence factors [30]. Our results show that the *perR* gene was more common in the strains isolated from women with puerperal fever than in the control group. Presence of PerR may be one reason for higher virulence of some GAS strains causing perinatal infections, but it certainly is not the only one. Uncontrolled and vast amounts of blood that are often present during labor in the uterus and surrounding organs and tissues, especially after cesarean section delivery, may allow the selection and fast growth of the strains that have the ability to bind and metabolize iron ions. Heme from red blood cells is the main source of iron for many pathogenic bacteria. As demonstrated recently by Sachla et al. [31], heme induces a broad stress response in GAS, and the success of GAS as a pathogen relies on mechanisms for heme sensing, detoxification, and repair. Thus, the mechanisms allowing its binding and absorption are potential targets for future prevention and treatment of the GAS infections.

One of the hypotheses on hyper-virulence of *S. pyogenes* leading to increased frequency of invasive infections concerns the presence of the genes encoding supertoxins in the genome of this bacterium [32]. This was not confirmed in this study, since there were no significant statistical differences between strains from invasive infections and those from unsymptomatic vaginal carriers.

The other hypothesis for the hyper-virulence of *S. pyogenes* is based on observations of increased frequency of digestive tract and vaginal group B streptococcus (GBS) colonization in pregnant women. Pregnancy may promote favorable conditions for GBS adherence in all of these ecological niches, where the epithelial cells possess the adequate receptor profiles. GAS has also been shown to possess an additional surface protein, *spy1325*, which structurally resembles the Rib protein present on GBS cells, and is described as having a strong affinity to vaginal epithelial cells [3]. This hypothesis was not confirmed by our study, which shows that the *spy1325* gene is significantly more common in isolates collected from women in the control group. The same is true for the *sla* gene, which was more common in the control group. The results of genotyping for GAS adherence genes were also confirmed by our phenotyping studies. Strong and intermediate adherence to a human cell line were significantly more common for strains originating from healthy vaginal microbiota than from puerperal fever or sepsis. It has been proposed on the basis of studies on pneumococci that effective adherence mechanisms are generally observed for bacterial strains causing infections originating from colonization of the host surfaces, rather than for highly invasive bacteria breaking host body barriers without a prior colonization phase [33]. Such a mechanism may be also true for hyper-virulent GAS strains causing sepsis or toxemia. The presence of certain genes coding for toxins was related to specific *S. pyogenes* serotypes, e.g., *speA* in *emm1* and *emm3*. On the other hand, *speC* was present in *emm2, emm28*, and *emm77* serotypes. The *sla* and *spy1325* genes were virtually absent in *emm1*, which was the second most common GAS serotype detected in the female genital tract. This observation may confirm the hypothesis that adherence properties are not important determinants of *S. pyogenes* invasiveness.

Our results indicate that that *S. pyogenes* strains related to invasive perinatal infections in women represent the *emm28* serotype (less commonly *emm1)* and possess mechanisms allowing the binding and metabolism of iron ions. It should be stressed that there are multiple other, yet unknown mechanisms, which may also help to explain the ability of *S. pyogenes* to cause invasive infections in postpartum mothers.
